# Acute Esophageal necrosis associated with diabetic ketoacidosis and alcohol intoxication

**DOI:** 10.1093/omcr/omaf265

**Published:** 2026-03-23

**Authors:** Arpit Shastri, K B Naveen, Arka De

**Affiliations:** Department of Hepatology, Post Graduate Institute of Medical Education and Research, Madhya Marg, Sector-12, Chandigarh 160012, India; Department of Hepatology, Post Graduate Institute of Medical Education and Research, Madhya Marg, Sector-12, Chandigarh 160012, India; Department of Hepatology, Post Graduate Institute of Medical Education and Research, Madhya Marg, Sector-12, Chandigarh 160012, India

## Case

**Figure 1:**
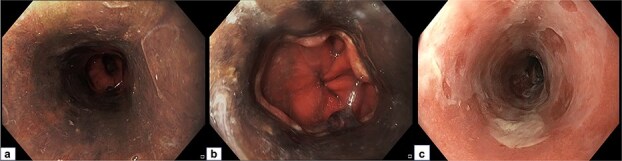
a: Diffuse circumferential blackening of entire esophageal mucosa. b: Distal esophagus showing sparing of gastroesophageal junction. c: Healed esophageal mucosa after 4 weeks.

A 52-year-old male presented with complaints of coffee-brown vomitus and melena for two days. There was no associated history of jaundice or abdominal distention. The patient had reported binge drinking few hours prior to his presenting complaints. Initial investigations revealed anemia (Hb: 10.2 g/L), deranged liver function parameters (total bilirubin: 3.8 mg/dL, aspartate aminotransaminase: 154 IU/L, alanine aminotransaminase: 60 IU/L) and high blood glucose levels (454 mg/dL) with detectable urinary ketones bodies and metabolic acidosis (pH:7.2). Upper gastrointestinal endoscopy revealed erosions and diffuse circumferential and necrotic blackening of entire esophageal mucosa which terminated abruptly at gastroesophageal junction. The gastric and duodenal mucosa were spared (Fig. 1a and b). Esophageal mucosal biopsies showed loss of surface epithelium with extensive necrosis in the lamina propria, thereby confirming the diagnosis of acute esophageal necrosis (AEN). The patient was managed with intravenous fluids, insulin, antibiotics and proton pump inhibitors (PPIs). After resolution of ketoacidosis and establishment of oral intake, the patient was discharged on oral pantoprazole and subcutaneous insulin. Repeat endoscopy after 4 weeks revealed significant healing of esophageal mucosa (Fig. 1c).

AEN or ‘black esophagus’ is a rare entity with a reported incidence of 0.008% to 0.2% [1]. Patchy or diffuse circumferential ‘black’ discoloration of esophageal mucosa which often terminates at gastroesophageal junction, with sparing of gastric mucosa is a characteristic feature. This is likely due to the increased susceptibility of ischemic esophageal mucosa to acid exposure. Risk factors include alcohol binging, diabetic ketoacidosis, connective tissue disorders, malignancy and fungal infections. Management is primarily conservative focusing on timely resuscitation, intravenous hydration, and PPIs. Ryle’s tube insertion is usually avoided for risk of perforation. As such, prompt and appropriate treatment of the underlying medical condition is the central pillar of management of acute esophageal necrosis [2, 3]. Mortality is uncommon and is usually due to the severity of underlying illness rather than esophageal complications like perforation [3].

## Guarantor

Arka De.
